# Gender Beliefs in the Kitchen: A Qualitative Exploration of Safe Food Handling Behaviours in Australia

**DOI:** 10.3390/bs16030447

**Published:** 2026-03-18

**Authors:** Nicolas La Verghetta, Matthew Phillips, Chloe Maxwell-Smith, Barbara Mullan

**Affiliations:** School of Population Health, Curtin University, Bentley, WA 6102, Australia; barbara.mullan@curtin.edu.au

**Keywords:** safe food handling, gender norms, reflexive thematic analysis, risk perception

## Abstract

Foodborne illness remains a persistent public health issue, yet domestic food safety practices are shaped by individual knowledge, social expectations, and gendered norms. This study examines how gender norms and expectations shape Australian consumers’ safe food-handling knowledge, perceptions, and practices. Guided by a social constructionist epistemology and feminist framework, semi-structured interviews were conducted with 28 participants aged 18–24 years recruited from a university research participation pool. Data were analysed using reflexive thematic analysis. Three themes were identified: “I know what I am doing”, optimism bias and false confidence, “Men’s casualness versus women’s strictness”, gendered safe food handling practices and expectations, and “Careful about others, relaxed for myself”, food safety as a social performance. Participants often expressed false confidence in their practices, reflecting optimism bias and reduced perceived susceptibility to foodborne illness. Women tended to portray vigilance and responsibility, while men described more relaxed approaches, reflecting gendered socialisation. Food safety also emerged as performative, with heightened care displayed when cooking for others. These findings highlight that domestic food safety is socially embedded and both reflects and reproduces gender norms. Addressing these dynamics through socially informed, context-sensitive interventions may improve public health outcomes.

## 1. Introduction

### 1.1. Safe Food Handling

Foodborne illness represents a substantial public health concern, resulting in 420,000 deaths globally per year, a statistic that continues to rise (Food Standards Australia, 2023). Foodborne illness affects 4.7 million Australians annually with 47,000 hospitalisations and 38 deaths each year (Food Standards Australia, 2023). However, these figures likely underestimate the burden, as most cases go unreported because individuals do not know where to report their illness (Elbehiry et al., 2023) or do not recognise their symptoms are food-related (Scallan Walter et al., 2021). While foodborne illnesses pose risks for everyone, specific populations face heightened vulnerability, including pregnant women, young children, older adults, and immunocompromised individuals (Centres for Diseases Control Prevention, 2019; Njoagwuani et al., 2023). Most instances of foodborne illness are sporadic, typically affecting individuals or small groups, which makes them less likely to be captured in government reporting systems (Redmond & Griffith, 2003). Additionally, foodborne illnesses cost Australia $2.4 billion annually due to medical expenses, reduced workplace productivity, and absenteeism (Food Standards Australia, 2022)

Many foodborne illnesses are caused by improper food handling practices, which can be prevented by adhering to food safety guidelines (Government of Western Australia Department of Health, 2020). Symptoms range from mild to severe, including diarrhoea, vomiting, headaches, abdominal cramps, and nausea, with some cases leading to chronic conditions or premature death (Lee & Yoon, 2021). The most frequent location for foodborne illness is the home (Charlesworth et al., 2021; Liguori et al., 2022). This finding contrasts with public perceptions, as individuals more frequently associate negative food experiences with venues outside the home (Zanetta et al., 2022). As the most frequent location for foodborne illness to occur is the home, discrepancy across contexts may reflect the evolution of the modern kitchen as a multifunctional space, where individuals do not always adhere to strict food safety guidelines expected in commercial food preparation settings (Food Standards Australia, 2023; Sanlier & Konaklioglu, 2012). In this setting, food preparation often co-occurs with caregiving, work, and leisure activities, which may reduce attention to food safety practices and encourage reliance on habits or informal heuristics rather than formal guidelines (Evans & Redmond, 2019). Individuals commonly report concerns about the hygiene standards of food outlets, as well as uncertainty or variation in appropriate food preparation and storage practices (Liguori et al., 2022). These perceptions may reflect broader issues regarding food safety awareness and access to reliable information. Beyond knowledge deficits, safe food handling within the home is influenced by psychological and social factors, including individuals’ perceptions of risk, habitual decision-making, confidence in food preparation, and socially learned norms regarding responsibility for food safety. These factors shape not only the likelihood of foodborne illness but also everyday preventive behaviours, learning pathways, and engagement with food safety guidelines (Charlesworth & Mullan, 2023; Levy et al., 2008).

Household food handling represents a modifiable and preventable site of foodborne illness risk, where individual knowledge, perceptions, and social roles directly influence food safety outcomes (Lopez Mato et al., 2022). Consumer knowledge regarding safe food handling remains low (Charlesworth & Mullan, 2023; Moreb et al., 2017; Mullan et al., 2015b), contributing to rising rates of household food poisoning (Charlesworth et al., 2021; Havelaar et al., 2015). Limited understanding represents one potential contributor to these unsafe practices and may be compounded by practical difficulties with food safety, including food affordability, varying access to quality produce, and food’s visual or aesthetic appeal (Behrens et al., 2010; Neve et al., 2021). Young adults (aged 18–24) represent a population at risk of non-adherence, as they are developing skills in shopping for and preparing food (Mullan et al., 2015b; van Rijen et al., 2021). Importantly, attitudes and the factors that shape them, including type of consumer, attitudes toward risk, and household food purchasing norms, may vary considerably within this demographic and influence adherence to safe food-handling practices (Czernyszewicz, 2023). Education about safe food handling practices can improve food safety behaviours, thereby reducing the short and long-term risks of foodborne illnesses (van Rijen et al., 2021). Evidence indicates that knowledge relating to food poisoning, personal hygiene, temperature control, and cross-contamination and cleaning remains limited among young adults, and strengthening these domains may help mitigate foodborne illness risk (Muhyaddin & Sabir, 2022). Understanding knowledge and practices related to food safety in young adults is essential for targeting future campaigns to address knowledge gaps and misconceptions about the causes of foodborne illnesses (Charlesworth et al., 2021).

### 1.2. Food Safety and Gender

In this context, gender is understood not simply as a demographic characteristic, but as a socially constructed set of norms, identities, and expectations that shape domestic roles, perceptions of risk, and responsibility for caregiving and food-related labour (Christopher, 2024). Food safety within the home includes cooking, cleaning, chilling, and storing food in ways that prevent foodborne illnesses (Charlesworth & Mullan, 2023). These tasks often fall disproportionately to a single individual in the household to adopt the “safe food handler” role, and this is frequently women (Das & Mishra, 2021). Domestic tasks related to food safety are often aligned with caregiving duties and risk management routines that have historically been gendered (DeVault, 1991). Consequently, women are socially positioned as being more attentive to cleanliness, organisation, and managing domestic risks, including food safety (He & Noussair, 2023). These gendered expectations may influence food safety outcomes through differences in perceived responsibility, vigilance, confidence, and accountability for preventing harm to others within the household. In traditional households, women continue to perform most of the domestic labour, even when employed outside the home, and remain primarily responsible for food safety-related tasks, including cooking, cleaning, and shopping (Milovanova et al., 2024). In contrast, men’s traditional household roles tend to focus on tasks that are less routine, more physically demanding, or occur less frequently, such as yard work, home repairs, or financial management (Christopher, 2024). This unequal distribution of food safety responsibilities may appear one-sided, with women assuming the majority of these duties, indicating the persistence of gender stereotypes and sociocultural patterns regardless of whether this division is intentionally established (Das & Mishra, 2021).

Gender stereotypes and social norms often limit women’s participation in food safety policymaking, despite their extensive household expertise that could inform more inclusive and effective regulations (Milovanova et al., 2024). Despite women’s important role in food safety, a significant research gap exists in understanding the intersection of food safety, gender, and social dimensions, particularly regarding the cultural norms that shape safe food handling practices and why these practices are excluded from public discourse. This research sought to explore young adults’ current understanding of safe food handling and the influence of gender experiences, norms, and identity on safe food handling perceptions. Understanding safe food handling requires attention not only to foodborne illness risk, but also to how individuals learn, interpret, and prioritise food safety practices within everyday domestic contexts. The aim of this study was to understand how Australian consumers’ safe food-handling knowledge, perceptions, and practices may be influenced by gender norms and expectations. This research may contribute to not only further educate individuals about correct safe food handling behaviours but also allow form a deeper understanding of how an individual’s gender identity may influence these behaviours.

## 2. Materials and Methods

### 2.1. Design

This study was intentionally exploratory in nature and was not designed to test, compare, or operationalize a specific theoretical model. Specifically, the study explores how gender norms and expectations influence safe food-handling knowledge, perceptions, and practices. By using an exploratory qualitative design, informed by a social constructionist epistemology and feminist theory. This study is underpinned by an interpretivist qualitative methodology, which seeks to understand how meanings and practices are socially constructed within specific contexts. Semi-structured interviews were conducted and analysed via reflexive thematic analysis. Consistent with this epistemological position, qualitative methods were selected to capture participants’ subjective accounts and the social contexts shaping food safety practices. Social constructionism epistemology posits knowledge and meaning as socially and contextually constructed (Phillips, 2023) and acknowledges the researcher’s active role in the analytic process (Braun & Clarke, 2021). Reflexive thematic analysis was therefore selected due to its alignment with a social constructionist epistemology, emphasizing researcher reflexivity and the interpretation of patterned meanings rather than the identification of objective truths (Braun & Clarke, 2021). A feminist theoretical perspective was applied to examine how food safety practices in the household both reflect and reproduce gender inequalities. This feminist perspective was integrated within the methodological approach to sensitize the analysis to power relations and the gendered organization of household food practices. Rather than applying a single feminist model, feminist theory was used as a sensitizing framework to attend to gendered norms, power relations, and the distribution of responsibility within household food practices (Kemmer, 2000). This design prioritized reflexivity, transparency, and contextual interpretation over evaluative judgements of correctness in food-handling practices

### 2.2. Positionality

As the lead author, I acknowledge that my positioning and unique experiences within the Australian higher education setting have influenced my approach to this research. It is also important to note, for the purpose of transparency, that I, the lead author, have over a decade of experience in the hospitality and restaurant industry, during which I completed multiple training related to safe food handling. The other authors on this manuscript have several years of academic experience, including one author with over two decades of expertise in food safety research and health psychology. My identity as a white male has been a focal point of discussion within the research team process. As a white, cisgender male researcher, I acknowledge the challenges I may face in fully representing and interpreting the experiences of women in relation to both their safe food handling behaviours and the gender norms that shape them. It is important to recognise the influence of traditional gender norms on these behaviours, and, as a man, I am committed to critically reflecting on my own positionality and continually acknowledging and accounting for potential biases throughout the research process., discriminatory assumptions, or the marginalisation of women in the framing of the “traditional safe food handler” role. With social constructivism and feminist theory guiding this research, I reflected on my thoughts and feelings and engaged in regular reflexive discussions with my supervisors to acknowledge the role of my gender identity and personal perspectives in shaping my interpretation of the data. Before the interviews, participants were informed that discussing gendered experiences might bring up unexpected emotions. They were reminded they could skip any question or stop the interview at any time. I occasionally shared my own gendered experiences to foster openness and reciprocity. As most participants were women aged 18–24, I recognised the gender and age difference between myself (a 29-year-old man) and the group. To address this, I focused on building rapport through empathetic listening, using inclusive language, and maintaining a respectful, non-authoritative tone. I also sought regular supervisor feedback to remain aware of and reflect on any unconscious gendered assumptions in my interactions and interpretations.

### 2.3. Participants

Young Australians, age 17 and older, were recruited using purposive non-probability sampling, from the university’s undergraduate research participation pool were recruited. Twenty-eight participants were interviewed (*M_age_* = 20, *SD* = 1.3; range = 18–24). Most identified as women (*N* = 20, 71%). A few participants reported having a food-related issue or allergen (*N* = 4) or having been hospitalised or treated with medication for severe foodborne illness (*N* = 2). Participant demographics are reported in Table 1. Sample size was determined using the concept of information power, which proposes that smaller samples may be sufficient when the study aim is narrow, the sample is specific, data quality is strong, and analysis is theoretically informed (Malterud et al., 2016). The present study had a clearly defined exploratory aim, a relatively homogenous sample of young Australian adults, and in-depth semi-structured interviews, supporting adequate information power with 28 participants. Data collection continued until sufficient informational redundancy was achieved, such that additional interviews no longer contributed substantively new insights relevant to the research aims. At this point, further recruitment was deemed unnecessary.

### 2.4. Materials

The semi-structured interview guide was developed based on the research aim and relevant literature and refined by the research team. The questions were open-ended, allowing for follow-up prompts to explore topics in more depth, uncover participants’ perspectives and underlying meanings, and foster rapport to understand their socio-cultural context (Abdul Majid et al., 2017). Before the interview, participants also completed a short demographic survey via Qualtrics, which included items on age, gender identity, experiences with food poisoning, and food-related health issues. These data were collected to contextualise the sample, and the survey took approximately five minutes to complete. The semi-structured interview contained 14 questions and was estimated to take 30 min. Questions were based on two research interests: (i) to explore individual safe food handling knowledge and behaviours (e.g., “Can you describe any training you have had in safe food handling?”, “how often do you prepare meals for yourself at home compared with using other food sources such as takeaway or food delivery services?”); and (ii) to explore if knowledge and behaviours were influenced by gender norms (e.g., “In the demographic survey you identified as man/woman, what do you believe are some of the norms or expectations surrounding safe food handling in relation to this gender identity?”). In certain instances, individuals who selected that they were hospitalized or medicated due to foodborne illness or had a food related allergen where also asked questions regarding those experiences (e.g., “Can you describe the time you were sent to the hospital because of your food poisoning? Does this experience affect how you interact with this food today”?).

### 2.5. Procedure

This research was approved by the university’s human research ethics committee (HRE2025-0206). Participants were provided with a participant information sheet, consent form, and demographic questionnaire before the interviews began. Semi-structured interviews were audio and visual recorded and conducted using Microsoft Teams, averaging 17 min (SD = 5.3 range 12–31 min) and transcribed verbatim. Interview transcripts were inspected for errors and cleaned prior to analysis. Participants were offered information regarding safe food handling behaviours upon interview completion. Software used to transcribe, and code, was Microsoft Word. Recruitment, data collection, and analysis occurred iteratively, with early findings informing the refinement of subsequent interviews and areas of inquiry.

### 2.6. Data Analysis

Reflexive thematic analysis was employed using Braun and Clarke’s (2019, 2021) guidelines. This method of analysis was chosen to assist in capturing the distinct experiences of young peoples’ safe food handling, and whether those experiences were motivated or influenced by aspects of their gender identity and norms. After the demographic survey, the interview took place. Following the interview, the transcripts were checked for consistency and transcription errors. Further immersion into the data involved multiple reviews and in-depth engagement with the cleaned interview transcripts. An inductive approach was useful in the development of early codes. These codes were further refined by the research team into categories, based on their relationships, resulting in the creation of distinct themes. Although coding was inductive, feminist and social constructionist perspectives informed how patterns of meaning were interpreted, particularly in relation to gendered expectations, responsibility, and everyday practices. These themes underwent iterative development, review, and finalisation through collaboration and discussion.

### 2.7. Quality

To support the rigour and trustworthiness of this study, I took several steps throughout the research process, including adhering to the guidelines set in the consolidated criteria for reporting qualitative research (Tong et al., 2007). Before conducting the full set of interviews, I piloted the guide with a small number of participants from trusted peers within our research group, to ensure questions were sensitive to the role of gender in everyday food handling practices. Prior to the interviews, all participants were informed that discussing their gendered experiences might evoke discomfort and they were able to skip questions or stop the interview at any time. I also shared some of my own gendered experiences where appropriate, to create an open and reciprocal space for dialogue. Throughout the study, I kept a reflexive journal of my thoughts, reactions, and assumptions to understand my own background (cis-gender, male) and beliefs around gender throughout my interpretation of the data. I met regularly with peers to talk through the coding process and the ideas emerging from the data. These conversations helped challenge my thinking, highlight alternative interpretations, and improve the overall depth of the analysis (Frost & Bailey-Rodriguez, 2019). To support transparency, I maintained an audit trail of coding decisions, theme development, and reflections. I fostered ethical sensitivity around this personal and culturally shaped topic by building rapport and creating a safe space for honest conversations.

## 3. Findings

Three themes were developed: “I Know What I Am Doing”—The Role of Optimism Bias and False Confidence in Safe Food Handling; “Men’s Casualness versus Women’s Strictness”—The Difference in Gendered Safe Handling Practices and Expectations; and “Careful About Others, Relaxed for Myself”—Food Safety as a Social Performance. These themes provide insight into how safe food handling knowledge and behaviours may be shaped by gender norms.

### 3.1. “I Know What I Am Doing”—The Role of Optimism Bias and False Confidence in Safe Food Handling

This theme explores participants’ knowledge of safe food handling as shaping their confidence in undertaking safe food handling behaviours in their household. Most participants were able to identify at least one domain of safe food handling, such as checking food date labels, using proper chopping boards, wiping down surfaces, or handwashing. These practices were learned from parents, secondary school cooking lessons, or knowledge gained in restaurant or café settings where they were employed. In these places of employment, food safety is enforced by health and safety regulators. Although participants had not received formal training, they reported relying on guidance from senior staff, who were assumed to have basic safe food handling knowledge, due to their seniority and experience in the workplace. *“I think at work our training is pretty strict? My manager does like to keep everything clean and keeping the environment just safe for food. We definitely go through a lot of training at work as well” (P1, Woman, 22).*

Despite awareness of safe food handling practices, participants often referred to everyday shortcuts, generally involving a visual or smell test. The “sniff test” was mentioned repeatedly to check whether food was safe to consume, as one participant explained: *“I don’t really pay attention to it much at home. If it looks good, smells good, it must be good” (P2, Man, 21).* For many, applying knowledge to prevent foodborne illness was not the primary concern; rather, their knowledge was used to create simple and convenient routines for preparing food in the household. When asked to rate their confidence in performing safe food handling practices, most participants gave themselves an eight or nine out of ten. This confidence stemmed from the view that food safety practices were straightforward and very simple to follow. As one participant explained, although she knew the rules, the absence of consistent consequences meant she did not always follow them: *“I’m aware of all the rules, but honestly, I bend them all the time—I’ve never gotten food poisoning, so I must be doing something right.” (P27, Woman, 21).* This participant described a tendency resembling optimism bias in their safe food handling practices, where the absence of negative consequences from neglecting such behaviours appeared to reinforce the belief that they were not doing anything wrong by ignoring them. This, in turn, appeared to reflect an overestimation of their body’s ability to prevent foodborne illness, suggesting that consistent safe food handling was seen as less necessary.

Further, safe food handling behaviours were often described as habitual, requiring little effort to perform. The more experience an individual had with a safe food handling practice, the more routine it became for them to perform this behaviour. Participants also referred to past experience as justification for continuing with less safe practices: *“I push the boundaries on like our expiration dates… nothing has ever happened, so that’s not that big of a deal” (P22, Man, 23)*. In such accounts, safe food handling knowledge and the intention to use food date labels correctly were present, but participants’ confidence and experiences appeared to coexist with a low perception of risk. The participant then discussed because of his lack of consequence, that they were much more likely to perform improper safe food handling, regardless of knowledge.

Overall, participants’ confidence in food handling appeared to stem from experiential learning through family and work contexts, rather than from formalised food safety knowledge. Many described feeling certain of their routines, even when some practices were not consistently safe. Participants often referred to shortcuts or familiar strategies, such as “cooking the same food every day” which required less cognitive effort in thinking about if they are performing safe food handling correctly. These accounts suggest that confidence was sustained by routine and prior outcomes, with limited attention to potential risks, highlighting the ways knowledge and practice can coexist with cautious or inconsistent adherence to safety guidelines or what they have learned previously. As a result of these routines and the absence of negative consequences, participants demonstrated an optimism bias in their approach to safe food handling.

### 3.2. “Men’s Casualness Versus Women’s Strictness”—The Difference in Gendered Safe Handling Practice and Expectations

This theme highlights the gender differences in practices and knowledge regarding safe food handling in the household. Participants, regardless of identity, described men traditionally as more casual and unconcerned, relying on quick judgements. Women however, were reported to be more strict and attentive to detail: *“He’ll just eat it if it looks okay, doesn’t even check the dates.” (P1, Woman, 22)* Women described themselves taking responsibility for correcting or compensating for male family members’ more relaxed behaviours. For some, this was linked to a sense of personal responsibility if something were to go wrong in their household, *“Even when I explain why, it doesn’t really change much. He (my brother) just says he’s never been sick, so he doesn’t see the point.” (P8, Woman, 20).*

Women’s vigilance surrounding safe food handling practices was not only about their own safety, but also protecting others in the household, discussing a sense of gendered patterns of responsibility. These women acknowledged that men often had less education about food safety than women but did not place blame or shame on their male partners or family members. As one participant explained, *“There’s not a set gender role, which I think is starting to slowly play an impact, but I definitely think that young guys are way less educated about it than the girls” (P9, Woman, 19).* Participants also acknowledged specific foods that were linked to gendered safe food handling practices, with these foods as exceptions to men’s casual approach to safe food handling. For example, chicken was important to men as it was critical to hit their protein goals for their workouts/gym routine, leading to greater awareness of the risks in handling and cooking it: *“Chicken’s the one food I think twice about, but apart from that I don’t really worry.” (P17, Man, 20).* All men discussed chicken with a degree of care; however, this concern was tied less to foodborne illness prevention or safe food handling and consuming more protein. This suggests that men became more invested in food handling when the perceived consequences were meaningful to them. In the case of chicken, both the risk of illness and the potential loss of valued protein appeared to create a stronger motivation to handle and prepare it carefully, regardless of quality or timing: “Leftovers (of chicken) are always fine for me: I’ve never gotten sick from them.”. Even if participants were aware of the guidelines for chicken and other meats, their responses indicated that strict adherence might not always occur.

Although men were aware of this difference in safe food handling, with at least half of the male participants acknowledging this difference and suggesting that they may need to change in the future if they wanted to be better role models for their children: *“When I’m a dad, I’ll have to be more careful, I don’t want my kids to get sick” (P17, Man, 20).* The prospect of becoming a parent or caregiver appeared to shift these patterns, with men recognising that their casual practices might no longer be acceptable when others’ well-being was at stake. For some, these expectations were tied to protecting children and family members. Women explained that they were more careful when cooking for others than when cooking for themselves, linking food safety to accountability and care. In doing so, they highlighted how women may carry not only the practical responsibility of safe food handling but also an emotional weight to be the best women they can be in the household. This highlights how caregiving roles introduced new expectations around responsibility and risk, suggesting that safe food handling practices were not fixed but responsive to changing household roles and relationships.

Participants also spoke to the constructed reality versus expectation of women’s safe food handling practices in the household. The shift from “domestic goddess” to equal role sharing in the household was evident, but women, described safe food handling as part of their everyday role within the household, reflecting unspoken expectations rather than explicit rules: “No one tells me to do it, I just always wash things down because that’s how I was taught.” (P21, Woman, 21) Women participants described that they never felt they were forced to perform these safe food handling behaviours based on their gender identity, but more as a sense of pride in what it meant to maintain a good household. These gendered expectations were internalised as part of being a “good” household food handler. In this way, women’s accounts highlighted how food safety practices intersected with broader ideas of gendered responsibility, distinct from the more casual approaches described by men. Additionally, women participants mentioned learning what it meant to manage a household properly from their mothers from a young age, *“It’s just part of keeping a proper house, my mum always said the kitchen shows what kind of person you are.” (P8, Woman, 20).*

Participants’ accounts highlighted gendered contrasts in safe food handling, where women positioned themselves as vigilant and accountable, while men were more casual and confident in taking shortcuts. At the same time, men recognised specific contexts where greater care was required, suggesting that their practices were not entirely fixed. These accounts also suggested that others did not explicitly expect women to take on this role. However, many had been taught from a young age that maintaining a good household meant being attentive to food safety. In this way, responsibility was internalised by women as part of their understanding of what it meant to manage a household properly. At the same time, men tended to take it up more selectively.

### 3.3. “Careful About Others, Relaxed for Myself”—Food Safety as a Social Performance

This theme explores the relational dimensions of food safety, where participants’ practices shift between careful orchestration when preparing food as an act of care for others, and more intuitive, risk-tolerant approaches when cooking becomes a private, self-directed activity. A participant summarised the distinction between preparing food for others, versus the self, and how safe food handling practices emerge as socially performative: “I don’t really wash the chopping board every time, unless someone else is watching.” (P21, Woman, 21). Such events were situational and contextual for participants rather than fixed. When cooking alone, many admitted to taking shortcuts or relaxing their standards, but when others were present, they described becoming more attentive to their safe food handling practices. “If I’ve got people over, I’ll be more careful, like making sure everything’s clean” (P16, Woman, 24). Together, these accounts highlighted how food safety practices were shaped by visibility and the need to maintain a certain impression in front of others. Beyond social impression, participants also described accountability within their own households. The social impression here is composited when participants framed food safety as part of being seen as a capable, responsible adult or household member. Doing things “properly” in front of others lets them be seen as competent, even if they cut corners alone. In this theme’s context, accountability was described as fragile and conditional, often heightened when others were present but fading when participants cooked alone. This distinction made accountability in this theme less about fixed “rules” or gendered practices, and more about the situational presence of others that created or undermined responsibility. Some explained that their influence was limited even when discussing food safety with others. For example, several described frustrations when partners dismissed their reminders, reflecting that accountability could be unevenly shared or even resisted. These accounts illustrated how negotiation and accountability were unevenly shared, with food safety practices sometimes undermined by others’ scepticism. Participants suggested this scepticism reflected power dynamics in shared spaces, where some household members dismissed food safety practices as unnecessary or excessive. Several noted that their reminders were rejected with comments like “I’ve never been sick” (P3, Female 19) or “someone else will do it” (P4, Man, 20), which positioned food safety as out of their control rather than essential. This highlighted how relational power shaped whether accountability was recognised or ignored.

At the same time, family roles, especially caring for children or younger siblings, introduced a stronger sense of responsibility. Several participants described being more vigilant with safe food handling practices when preparing meals for others. One participant explained, “When it’s for my younger siblings I cook for, I’m more strict with dates… For myself I wouldn’t bother…” (P19, Woman, 18). Here, food safety was framed as a protective responsibility, with children’s well-being elevating the importance of care. Some also noted that while they relaxed standards with friends or for themselves, children were a “non-negotiable” context where safety rules had to be followed. Participants contrasted their relaxed approach when cooking for themselves or friends with stricter vigilance for children, describing children as a “non-negotiable” context where mistakes felt morally unacceptable. In this way, responsibility was described less as gendered and more as a moral threshold tied to protecting dependents.

Overall, participants presented food safety as both practical and performative. While personal routines often involved shortcuts, the involvement of others introduced expectations of care, responsibility, and accountability. These accounts suggest that food safety was not only about individual knowledge or gendered responsibility but was also relational, shifting depending on who was present and what was at stake.

## 4. Discussion

This study explored how Australians navigate safe food handling, with particular attention to how gender norms and expectations shape knowledge, perceptions, and practices. Confidence within the participants reflected an optimism bias in which participants underestimated their vulnerability to foodborne illness. Men tended to associate safety with resilience and toughness, consistent with cultural ideals of self-reliance, while women grounded their confidence in experience and sensory cues such as smell or appearance heuristics that overlook the invisible nature of pathogens. Food safety responsibilities were unevenly distributed, with women commonly positioned as the default household risk managers. Participants also described adjusting their practices in social settings to display competence and care. These performances reinforced gendered expectations, situating women as primary caregivers and linking food safety to emotional and moral ideals of being a “good” and responsible household member.

The gendered patterns observed in this study are consistent with those reported in previous research. In household settings, women are more likely than men to assume responsibility for food preparation and food safety practices (Lopez Mato et al., 2022; Milovanova et al., 2024). Quantitative studies have also identified lower perceived susceptibility and greater optimism bias among men in relation to foodborne illness risk (da Cunha et al., 2015). The present qualitative findings extend this literature by illustrating how these patterns are reinforced through gender norms and expectations, offering contextual insight into the social mechanisms underlying observed behavioural differences.

Participants described strong confidence in their food handling practices, grounded in personal routines rather than formal guidelines. Many assumed their methods were safe because they had rarely experienced noticeable illness, reflecting optimism bias underestimating susceptibility to foodborne illness while overestimating ability. Instead of recognising food safety as an ongoing risk, participants positioned themselves as competent even without accurate knowledge. This misplaced confidence encouraged behaviours that increased risk and discouraged behavioural change, maintaining a cycle of optimism bias that could be passed on to others. Participants often downplayed handwashing, food storage, or checking date labels, justifying omissions because their approach “had always worked.” Minor symptoms such as stomach upset were dismissed as unrelated to food preparation, reinforcing the belief that their behaviours carried little real risk. In this way, optimism bias acted as a protective narrative, allowing risky practices to continue without consideration of potential consequences. These findings highlight the need for intervention-based practices of safe food handling that target risky behaviours and individuals’ optimism bias. Programs must go beyond providing guidelines and instead reveal the gap between perceived and actual competence. For men, strategies could include social comparison or humour that challenges toughness as protection (Budden et al., 2022). For women, targeted educational interventions have been shown to be the most effective method for improving safe food handling practices, as they are generally more receptive to food safety education, especially during significant life stages such as pregnancy (Kendall et al., 2017).

Over time, food safety became normalised as a routine task rather than recognised as a potential risk point. Unsafe practices were considered harmless if they had “worked before,” positioning risk as theoretical rather than personally relevant. These findings align with evidence that foodborne illness often goes unrecognised in the home (Scallan Walter et al., 2021) but extend existing work by showing that misplaced confidence, rather than lack of knowledge alone, sustains unsafe practices. Confidence without competence emerged as equally problematic, suggesting that awareness of guidelines may not be sufficient to shift behaviour. Knowledge of safe food handling has not always resulted in behaviour change, as the knowledge–behaviour gap is evident throughout safe food handling research (Young & Waddell, 2016). When individuals believe they already manage food safely, they are less likely to seek feedback, scrutinise routines, or adopt new precautions. This misplaced assurance creates hidden risk: people who feel capable may engage in more risky behaviours, such as skipping handwashing or ignoring storage temperatures because precautions are perceived as unnecessary.

Interview narratives highlighted food safety practices as shaped by household gender norms. Men were often portrayed as casual and unconcerned, relying on visual checks or personal judgment rather than formal standards. Women were characterised as stricter and more attentive, frequently taking responsibility for monitoring leftovers, checking date labels, and cleaning surfaces. These contrasts reflected behavioural differences and the gendered expectation that women bear primary accountability for household health. Similar findings have been presented in previous research in which women are more vigilant and practical than men (Fein et al., 2011; Sanlier, 2010). Previous work suggests that women’s vigilance is linked to stronger application of food safety knowledge, while men are more engaged in the food sector such as restaurants and catering industry (Sanlier, 2010). Fein et al. (2011) argue that women’s heightened responsibility stems from their role as primary caregivers. However, the findings here move beyond these explanations by showing that vigilance is not simply a product of caregiving roles. Instead, women constructed and maintained food safety behaviours through shared understandings of what it means to be a competent food handler at home. Women’s vigilance was not only about performing expected roles but about negotiating standards of competence and responsibility within the household—a nuance earlier studies did not capture.

Men’s accounts offered a nuanced perspective that both reinforced and challenged existing literature. Consistent with research showing men’s greater reliance on convenience and risk-taking (Redmond & Griffith, 2003), men often described casual food preparation practices. However, several participants showed willingness to adapt when circumstances demanded greater responsibility, such as preparing food for children or handling high-risk items, like chicken. This conditional engagement suggests men’s food safety behaviours are context-dependent, shifting in response to relational roles and perceived stakes. Men became most attentive when food safety intersected with personal health priorities, such as protein intake, or when caring for dependents. This indicates that men prioritise risks framed in terms of direct, tangible consequences rather than general household responsibility. While previous research has shown that context can shape food handling in institutional settings (Rustiawan & Suryani, 2021), this study suggests that in domestic contexts, men’s vigilance is selectively mobilised when risk feels personally consequential. For many women, vigilance in food handling was tied to protecting others and maintaining a well-run household.

Participants described learning these practices from their mothers and internalising them as part of what it meant to be a “good” food handler. These norms were transmitted intergenerationally and reinforced through daily practice, becoming naturalised over time. Men, by contrast, framed food handling as less central to their household role, becoming attentive only when risks threatened their own priorities. This contrast highlights how gendered divisions of labour persist: women’s practices are framed as care, while men’s engagement is legitimised as self-interest. This pattern aligns with DeVault (1991) analysis of feeding the family as a form of moral and invisible domestic labour, in which women assume responsibility for household food practices not through explicit negotiation but through taken-for-granted expectations of care and competence. Unequal responsibility for household food safety is maintained not through imposition but through alignment of social norms with personal identity. These findings reinforce prior evidence that women disproportionately shoulder the invisible labour of household food safety (Milovanova et al., 2024) and extend it by highlighting how men’s engagement fluctuates with context and relevance. Women’s concerns were sometimes dismissed by male partners, allowing men to downplay norms without consequence. These dynamics show that domestic food safety remains deeply entwined with gender and care, with women’s responsibility sustained by norms of care and men’s ability to reject them.

Food safety emerged as more than a matter of individual competence or technical compliance. Participants described how their behaviours shifted depending on social context, becoming more careful when others were present. Food preparation in these situations functioned as impression management, consistent with Goffman’s (1990) distinction between front stage and backstage. In “front stage” contexts, where food handling was visible, vigilance became a performance of competence, care, and responsibility. Extending this analysis, Butler (1988) concept of performativity highlights how such repeated practices do not merely express gender norms but actively constitute them over time. Through the routine repetition of vigilance, women’s careful food handling becomes part of what it means to enact a responsible feminine identity, while men’s selective engagement reproduces norms that position food safety as optional rather than obligatory. Conversely, in the “backstage” of cooking alone, participants admitted to shortcuts and intuition. Habits played a central role in shaping these patterns, creating a baseline of practice that felt adequate in familiar contexts, but the presence of others prompted stricter adherence to guidelines. In this sense, food safety was contingent, rather than consistent, reflecting the situational weight of habit and social expectation (Veflen et al., 2020). Previous work suggests that women’s vigilance is linked to stronger application of food safety knowledge, while men are more represented in professional food roles, such as chefs and restaurant staff. Gender shaped not only the degree of carefulness but also the performance of food safety across these contexts. Women described attentiveness as expected both privately and publicly, suggesting that their “front stage” extended into everyday domestic life. Men reported more freedom to treat food safety as a backstage concern, engaging selectively and often only when visible to others or when personal priorities were at stake. Thus, men and women differed not just in attentiveness but in how they performed safety for different audiences, women bearing continuous accountability, men performing selectively.

Several participants described children as a “non-negotiable” category for whom vigilance was essential, framing food safety as an ethical duty rather than preference. This suggests that food safety crosses a moral care threshold when dependents’ wellbeing is at stake (Mullan et al., 2015a), echoing ethics-of-care perspectives that link responsibility to relational obligations. Food safety was elevated from routine to protective care, showing how moral responsibility shaped behaviour. However, as previously noted, optimism bias and false confidence may still limit effective care. Areas of improvement were often evident among younger siblings (Meysenburg et al., 2014; Rheinländer et al., 2008). These accounts illustrate that food safety is not merely a technical skill but a social performance, enacted through visibility, shaped by habit, and reinforced by moral care. These findings build on prior work identifying food preparation as moral and social identity (Mullan et al., 2015a) extending this by showing how food safety becomes a site of social performance. Safety practices were about reducing risk and signalling competence, care, and respect for others. Interventions framed around protecting vulnerable family members or avoiding embarrassment may therefore resonate more strongly than those focused solely on individual health.

### 4.1. Practical and Theoretical Implications

Educational and government programs should consider the social and relational contexts of household food handling, recognising how practices are shaped by gendered expectations and different household roles and relationships. Messaging that accounts for social influence and responsibility may assist with this idea. Theoretically, this research demonstrates the value of feminist theory in understanding how food safety practices are socially constructed and embedded within household power structures. While integrating a social constructionist epistemology allowed for the identification of contextual meanings that guide behaviours, highlighting the importance of gender-sensitive underpinnings in exploring safe food-handling behaviours. These insights underscore the potential of combining qualitative methods with feminist perspectives to reveal the mechanisms through which social norms, expectations, and household roles and responsibilities influence safe food-handling practices.

### 4.2. Strengths, Limitations and Future Directions

One of the main strengths of this study lies in its exploratory qualitative approach, which provided rich and detailed insights into the lived experience of food safety within the home. Rather than treating unsafe practices as the outcome of knowledge deficits alone, the analysis illuminated the psychological and social mechanisms that underpin everyday behaviours, such as optimism bias, social performance, and gendered expectations. This perspective allowed for a more nuanced understanding of why individuals continue to engage in risky practices even when aware of guidelines, and how responsibility for safety is negotiated within households. The use of reflexive thematic analysis further strengthened the study by enabling an interpretive rather than purely descriptive account (Braun & Clarke, 2019), drawing attention to the ways in which participants constructed meaning around competence, responsibility, and care. The research contributes to a growing work that situates food safety within broader social and behavioural contexts.

While the study employed multiple strategies to ensure rigour and trustworthiness, certain limitations warrant consideration. The participant group was predominantly tertiary-educated, young adults, which narrows the scope of insights to a specific demographic still developing independent food safety routines. Although high educational attainment may influence participants’ risk perception and knowledge, this focus is appropriate for exploring early adult behaviours as they transition to independent food handling. This age-specific focus limits the transferability of findings to other life stages, such as older adults, parents, or individuals with greater caregiving responsibilities, who may approach food safety in distinct ways. Consistent with previous research involving university student samples, women were more highly represented than men in this study (Senn & Desmarais, 2001), which may have limited the representation of men’s first-hand perspectives. However, many women participants described men’s safe food-handling practices based on direct household and relational experiences. These accounts offer contextual insight into gendered practices and complement men’s self-reported experiences within the broader analysis. Self-reported accounts also pose challenges, as participants may have unintentionally minimised risky behaviours or emphasised positive practices due to pressures from social desirability during the interview.

Future research should broaden the demographic focus to capture how food safety practices differ across life stages, including older adults, parents, and individuals with ongoing caregiving responsibilities. Examining these groups would provide insight into how responsibilities for food safety shift as roles and household structures change. Interventions could also be refined by addressing optimism bias directly, testing whether messages framed around protecting others or men avoiding embarrassment resonate more strongly than those centred on individual health risks. Additionally, studies with a more gender-balanced sample, including greater representation of men, may further strengthen understanding of how gender norms shape food safety knowledge and behaviours. At the policy and education level, campaigns may benefit from moving beyond technical compliance and instead acknowledging food safety’s social and relational dimensions, positioning it as both a health practice and a marker of responsibility within the household. Finally, future studies should continue to examine how gendered practices shape the distribution of food safety responsibilities, as traditional caregiving roles remain highly influential in determining who performs and prioritises safe food handling.

## 5. Conclusions

This study examined how Australians navigate domestic food safety, showing that practices are shaped less by technical knowledge than by optimism bias, gendered norms, and social performance. Findings extend prior work by revealing that misplaced confidence whether grounded in stoicism, sensory familiarity, or routine sustains risky behaviours despite awareness of guidelines. Gendered divisions of responsibility persisted, with women naturalising accountability as part of being a “good” food handler. Food safety also emerged as performative, shifting across frontstage and backstage contexts, and crossing moral care thresholds when dependents were involved. These insights position food safety as a gendered social practice, contingent, moralised, and performed. Interventions should therefore move beyond knowledge provision to challenge misplaced confidence, disrupt unequal accountability, and frame food safety as a shared responsibility. Future research should examine these dynamics across diverse households and life stages.

## Figures and Tables

**Table 1:** Participant Characteristics.

	*n*	%
Gender Identity		
Woman	20	71.4
Man	8	28.6
Hospitalized/Medicated due to Foodborne Illness		
Yes	2	7.2
No	26	92.8
Food-related Allergen		
Yes	4	14.3
No	24	85.7

## Data Availability

The data presented in this study are available on request from the corresponding authors.
